# Interference between immune cells and insomnia: a bibliometric analysis from 2000 to 2023

**DOI:** 10.3389/fneur.2025.1486548

**Published:** 2025-03-26

**Authors:** Nana Tang, Yingjian Zeng, Guilian He, Shupeng Chen

**Affiliations:** ^1^Affiliated Hospital of Jiangxi University of Traditional Chinese Medicine, Nanchang, China; ^2^School of Clinical Medicine, Jiangxi University of Chinese Medicine, Nanchang, China

**Keywords:** insomnia, autoimmune encephalitis, natural killer cells, T cells, B cells, comorbid

## Abstract

**Background:**

Insomnia is a widespread sleep disorder that significantly affects the quality of life and contributes to immune dysfunction, which in turn leads to chronic diseases. Despite extensive research on sleep disturbances and immune modulation, the relationship between insomnia and immune responses remains underexplored.

**Objectives:**

The primary objective of this study was to conduct a bibliometric analysis to explore the interaction between immune cells and insomnia, identifying key immune responses involved and their potential roles in the development of insomnia and associated comorbidities.

**Methods:**

A bibliometric analysis was conducted using data from the Web of Science Core Collection (WoSCC), focusing on research articles published between 2000 and 2023. The analysis aimed to identify trends, key research areas, and the role of immune system cells (T cells, B cells, NK cells, etc.) in insomnia.

**Results:**

The analysis revealed that various immune cells, including T cells, B cells, NK cells, neutrophils, and monocytes, play crucial roles in insomnia pathogenesis. These immune cells contribute to immune modulation and inflammatory responses, which are linked to sleep disturbances. The study also identified that insomnia is closely associated with comorbidities such as cardiovascular diseases, obesity, depression, and cancer, all of which involve immune dysfunction. The regulation of the immune system was found to be a key factor in improving sleep quality.

**Conclusion:**

This study provides valuable insights into the complex interaction between the immune system and insomnia. The findings underscore the importance of immune regulation in the treatment of insomnia, suggesting that future research should focus on integrating immune modulation into therapeutic strategies for insomnia. Further studies are needed to explore targeted therapies for immune-related insomnia and its comorbidities, emphasizing interdisciplinary research in this area.

## 1 Introduction

Insomnia is a major global health challenge, affecting approximately 30% of the population with varying degrees of severity, and around 10% suffering from chronic insomnia (1). Not only does insomnia significantly impair individuals’ quality of life, but it is also closely associated with various physical and mental health issues (2). Current treatments for insomnia primarily include pharmacotherapy, such as benzodiazepines and non-benzodiazepines, cognitive behavioral therapy for insomnia (CBT-I), and alternative therapies like acupuncture and herbal treatments (3–5). However, these treatments have certain limitations. For instance, pharmacotherapy may lead to dependency and side effects, while CBT-I requires high patient compliance and efficacy. Additionally, insomnia is often comorbid with various conditions such as depression, hypertension, diabetes, cardiovascular diseases, and cancer, which increases the complexity and challenges of treatment (6–8). For example, the incidence of insomnia is significantly high among patients with depression, and conversely, insomnia may exacerbate depressive symptoms (9). This bidirectional relationship suggests that insomnia is not only a comorbidity of these diseases but may also be a contributing or aggravating factor. Therefore, it is crucial to explore the pathological mechanisms of insomnia, particularly its role in multisystem comorbidities, to develop more effective treatment strategies.

In recent years, the bidirectional role of immune cells in insomnia has garnered widespread attention. On the one hand, studies have shown that insomnia can trigger abnormal immune responses, such as increased pro-inflammatory cytokines and dysregulation of immune function (10); on the other hand, insomnia may exacerbate its course by affecting immune cell function. For example, sleep deprivation leads to an increase in the number of natural killer cells (NK+) and B cells (CD45+), while the number of cytotoxic T cells decreases, indicating that the immune system may play a critical role in the onset and progression of insomnia (11). As research progresses, the role of immune cells in insomnia and multisystem comorbidities is gradually being elucidated, highlighting their potential importance in regulating insomnia-related inflammatory responses and disease progression.

Bibliometrics, as a discipline that uses mathematical and statistical methods to analyze scientific literature, aims to reveal the dynamics of scientific research, research hotspots, and interdisciplinary integration by evaluating and quantifying the distribution, structure, growth, and interrelationships of the literature (12). Through this approach, researchers can assess research activities and influence in specific fields or topics, identify key journals and literature, and track scientific collaboration networks. Visualization methods, on the other hand, represent complex datasets graphically, making patterns and trends in the data more intuitive and understandable. Therefore, this paper aims to systematically review the research progress of immune cells in insomnia using bibliometric and visualization techniques, deeply exploring and analyzing the potential information in this field to provide valuable references for future research.

## 2 Materials and methods

### 2.1 Data source and literature search

To ensure the collection of high-quality, comprehensive, and standardized literature data, the Web of Science Core Collection (WoSCC) was selected as the primary data source. The literature data were retrieved from WoSCC, covering the period from January 1, 2000, to December 31, 2023. The search strategy employed the following query: [TS = (“innate immunity” OR “innate immune cells” OR “macrophages” OR “neutrophils” OR “monocytes” OR “dendritic cells” OR “natural killer cells” OR “NK cells” OR “eosinophils” OR “basophils” OR “mast cells” OR “adaptive immunity” OR “adaptive immune cells” OR “T cells” OR “B cells” OR “antibodies” OR “immune response” OR “immune system”)] AND TS = (insomnia OR “sleep disorders”).

### 2.2 Data screening

#### 2.2.1 Inclusion criteria

(1) Literature related to Research on Immunity and Insomnia; (2) Literature published in English; (3) Literature types include clinical trial studies, *in vitro* experimental studies, *in vivo* experimental studies, public database analysis studies, reviews, etc.; (4) Literature with complete bibliographic information(including title, country, author, keywords, source).

#### 2.2.2 Exclusion criteria

(1) Conference papers, newspapers, patents, achievements, health and popular science literature, etc.; (2) Duplicate publications; (3) The literature cannot be fully obtained.

The inclusion and exclusion process is independently conducted by two reviewers. If the inclusion and exclusion results are inconsistent, the third reviewer will participate in the work.

#### 2.2.3 Data standardization

After screening, the literature was exported in Refworks and plain text formats. Special symbols were removed. Keyword names were standardized, for example, “lack of sleep” or “poor sleep quality” should not be used; instead, the standard term “sleep disorder” should be uniformly adopted to cover all related sleep problems. Country/Region names were standardized, for example, “north ireland,”“wales,”“england,”“scotland” were determined as “united kingdom.” Then, the Data Import/Export function in CiteSpace software was used to convert the format of the retrieved literature.

#### 2.2.4 Data analysis

##### 2.2.4.1 Data extraction

The normalized text data will be incorporated into a structured form designed by two researchers, who will then extract relevant data. The extracted data includes the following parts: publication information, encompassing the year of publication, country/region, issuing organization, issuing journal, authors, cited literature, and keywords.

##### 2.2.4.2 Analysis methods

**Figure 1:**
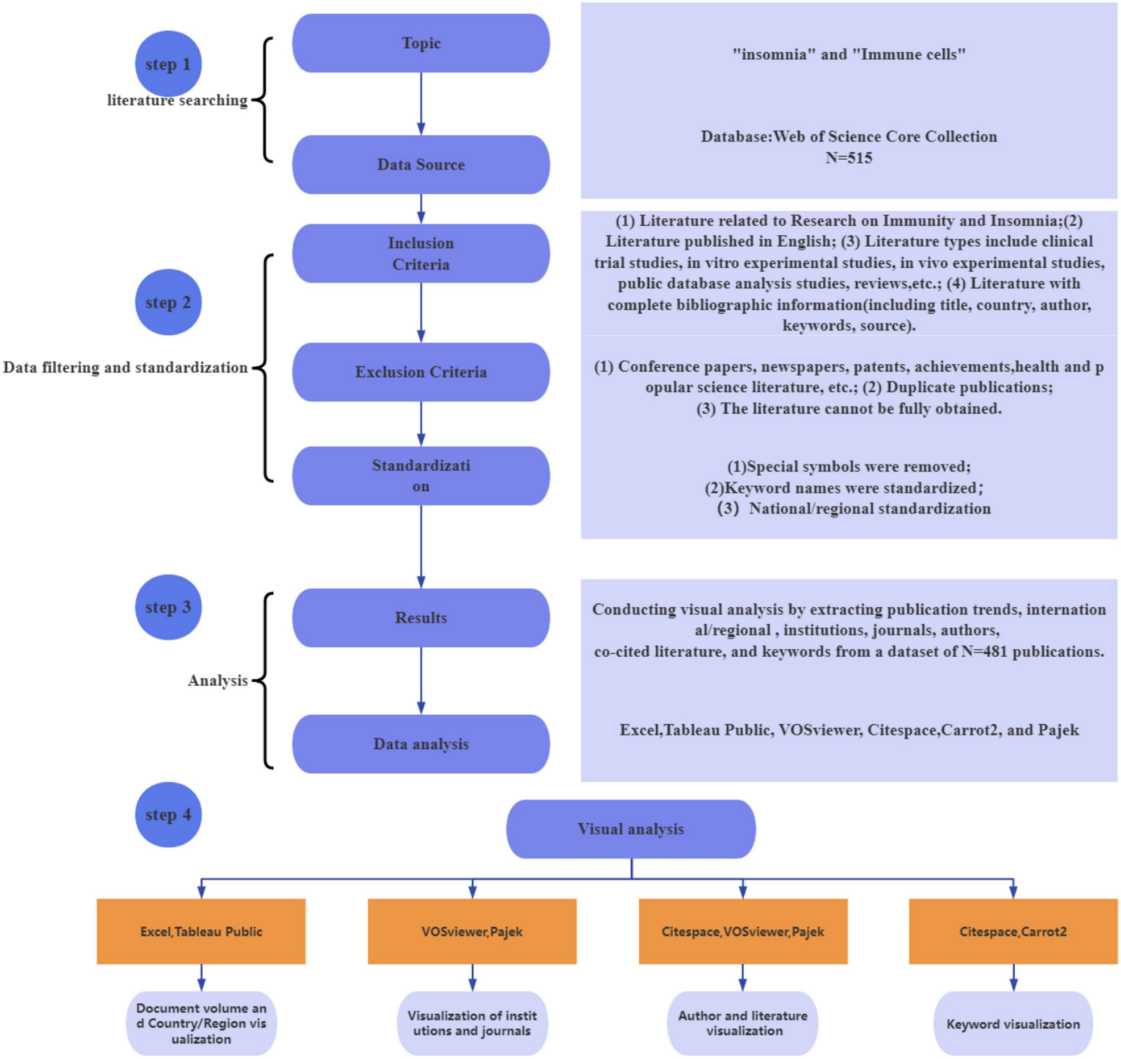
Visualization analysis process of immune cells and insomnia research.

This study employs bibliometric visualization analysis to systematically review and uncover valuable hidden information in this field. The visualization of publication volume was achieved by converting text data through CiteSpace’s data export to Excel, followed by the creation of fitted curves to accurately predict future publication trends. The visualization of countries/regions was carried out by converting text data from VOSviewer to Excel and then further processed using Tableau Public. Institutional visualization was achieved through a combination of VOSviewer and Pajek. The data analysis of journals and authors was conducted by exporting CSV files from VOSviewer based on the text files. Keyword visualization was performed by converting text data into XML files via CiteSpace and then generating bubble charts using Carrot 2. The co-cited references were visualized using the Bursts function in CiteSpace. The process of data acquisition and analysis is illustrated in Figure 1. The final set of included literature for analysis is provided in Appendix 1.

## 3 Results

### 3.1 Analysis of publication volume

**Figure 2:**
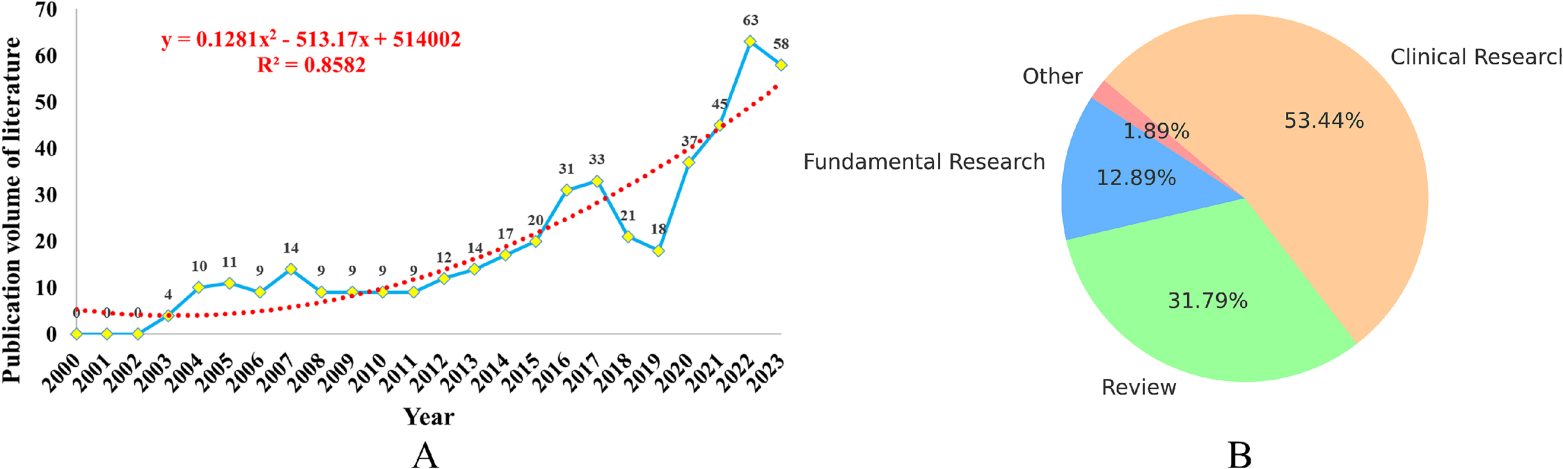
Analysis of the publication volume of research on immune cells and insomnia. **(A)** Trend line chart of publication volume. **(B)** Article classification pie chart.

In the past 20 years, the number of publications related to insomnia and immunity has steadily increased, as shown in Figure 2A. Since the first related reports emerged in 2003, the number of publications has continuously climbed. Notably, between 2003 and 2017, the number of studies in this field continued to rise. However, in 2018–2019, there was a slight dip in the number of publications. This decline could be related to the emergence of new research areas, such as the interaction between the brain and immune system, and the relationship between the microbiome and immunity, which attracted considerable attention from researchers, leading to a temporary cooling of research on the link between insomnia and immunity.

Since 2019, the number of publications related to insomnia and immunity has rapidly increased, which may be closely associated with the outbreak of the COVID-19 pandemic. The pandemic sparked greater focus on the immune system, depression, insomnia, and other neurogenic diseases, further promoting research in this field. By 2022, the number of related publications had reached 63 per year. Of these, clinical studies accounted for 53.40%, reviews made up 31.80%, and fundamental research represented 12.90%, as shown in Figure 2B.

To accurately predict future trends, we used a polynomial fitting curve (red dashed line) to show the ongoing growth of literature in this field. The coefficient of determination (R^2^ = 0.8582) indicates that this model can explain 85.82% of the data variability, providing a strong predictive reference value. This underscores the increasing importance of immunity in insomnia research, and highlights the promising future of this field.

### 3.2 Countries/regions

**Figure 3:**
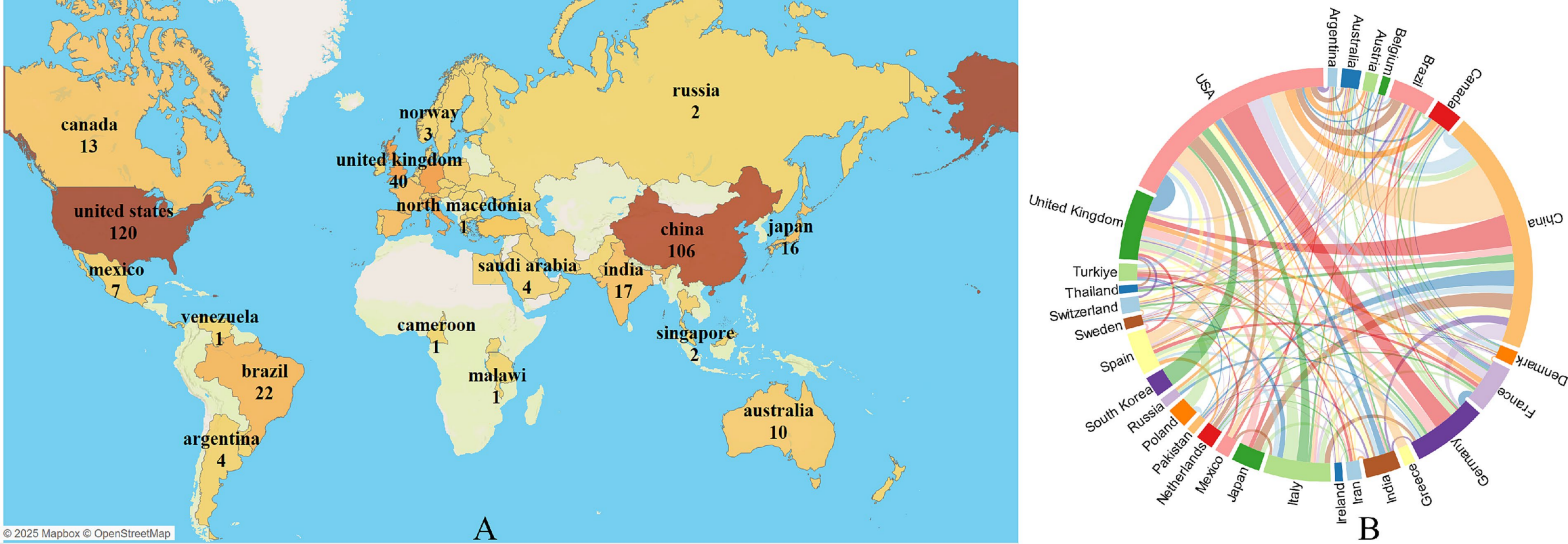
Visualization of immune cells and insomnia research by country/region. **(A)** Global geographic visualization. **(B)** Global cooperation string chart, with nodes representing the number of publications and lines representing the strength of cooperation.

During this period, authors from 62 countries/regions published their research findings. Figure 3A presents the geographic visualization of global publications related to insomnia and immunity. The United States is the leading country in terms of publication volume (*n* = 120), followed by China (*n* = 106), Germany (*n* = 40), and the United Kingdom (*n* = 40). Figure 3B displays a chord diagram of international collaboration, where the United States (*n* = 87) has the highest collaboration intensity, followed by Germany (*n* = 51) and the United Kingdom (*n* = 45). Table 1 provides a detailed description of the top countries in terms of publication volume, cooperation intensity, total citations, and average citations per paper. The United States has the highest total citations at 6,885, with an average citation of 57.375 per paper, followed by the United Kingdom (*n* = 2,145) and Italy (*n* = 1,740). Among the top 10 countries in terms of publication volume, eight countries—United States, China, Germany, United Kingdom, Italy, Brazil, France, and Spain—have more than 500 total citations. The geographic distribution of the literature indicates that research on the immune aspects of insomnia has garnered worldwide attention and holds significant influence.

**Table 1:** The top 10 countries according to the total publications during 2000–2023.

Rank	Country	Publication	Cooperation intensity	Total citations	Average citation per paper
1	United States	120	87	6,885	57.375
2	China	106	22	1,384	13.0566
3	Germany	40	51	1,596	39.9
4	United Kingdom	40	45	2,145	53.625
5	Italy	34	22	1740	51.1765
6	Brazil	22	17	679	30.8636
7	France	19	21	972	51.1579
8	Spain	19	20	860	45.2632
9	India	17	15	303	17.8235
10	Japan	16	8	497	31.0625

### 3.3 Institutions

**Figure 4:**
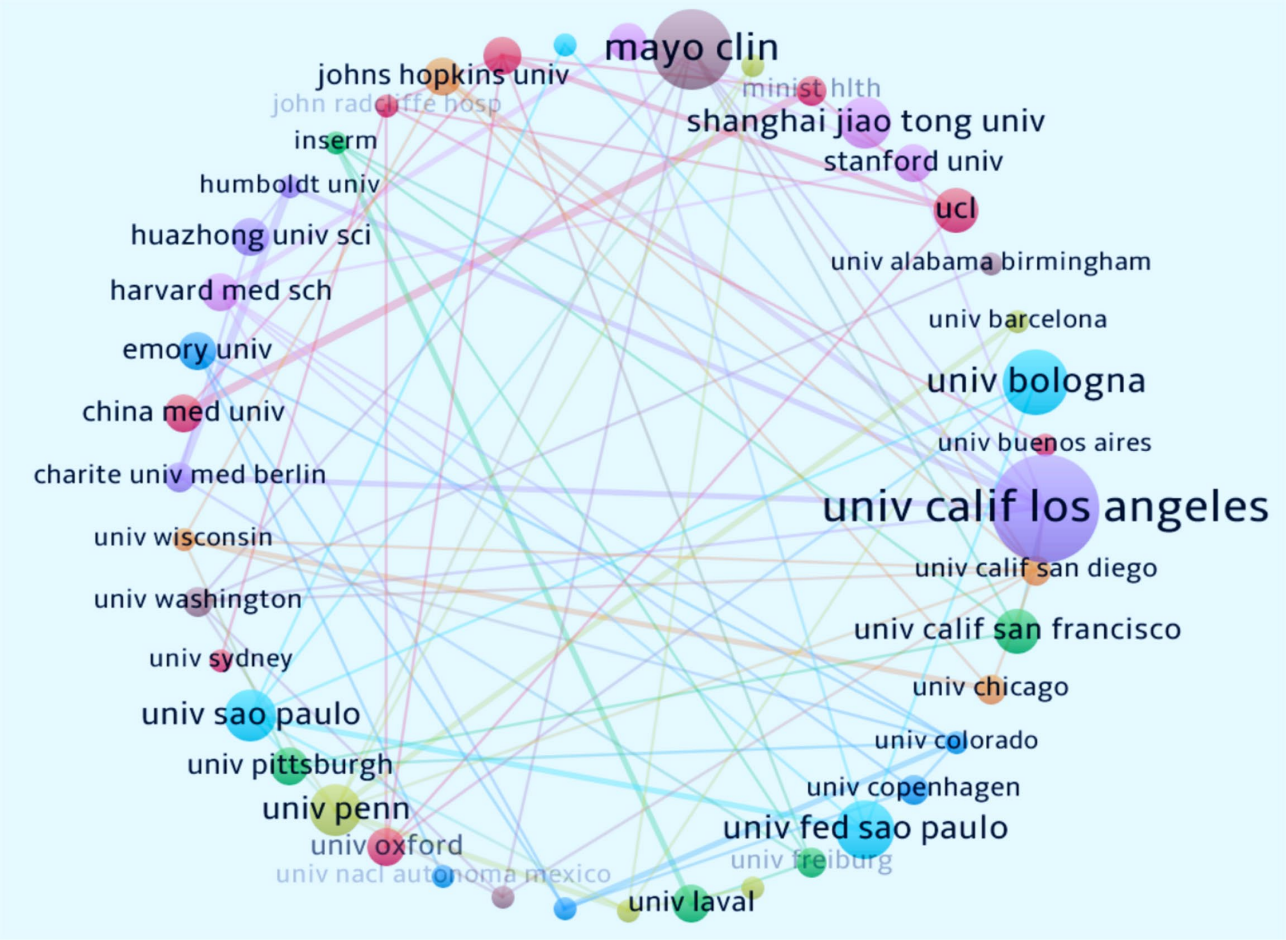
Collaborative network of institutions on immune cells and insomnia research. Nodes represent the number of articles published, while lines represent the strength of cooperation.

Over the past 20 years, 945 institutions worldwide have published research findings on the relationship between immunity and insomnia. Figure 4 illustrates the global institutional collaboration network in this field. The University of California, Los Angeles (United States, *n* = 15) is the leading institution in terms of publication volume, followed by the Mayo Clinic (United States, *n* = 11) and the University of Bologna (Italy, *n* = 9). The institution with the highest collaboration intensity is also the University of California, Los Angeles (United States, *n* = 12), followed by the University of Pennsylvania (United States, *n* = 10). Table 2 provides detailed information on the top 10 institutions, including publication volume, cooperation intensity, total citations, and average citations per paper. The University of Pennsylvania (United States, *n* = 107.8571) has the highest average citations per paper, followed by the University of California, Los Angeles (United States, *n* = 24.01). The collaboration among these institutions has significantly contributed to the development and dissemination of research in this area.

**Table 2:** The top 10 institutions according to research on immunity and insomnia during 2000–2023.

Rank	Institution	Location	Publication	Cooperation intensity	Total citations	Average citation per paper
1	University of California, Los Angeles	United States	15	12	1,571	104.7333
2	Mayo Clinic	United States	11	7	726	66
3	University of Bologna	Italy	9	3	185	20.5556
4	Federal University of São Paulo	Brazil	8	5	237	29.625
5	Shanghai Jiao Tong University	China	7	1	162	23.1429
6	University of Pennsylvania	United States	7	10	755	107.8571
7	University of São Paulo	Brazil	7	7	345	49.2857
8	University College London	United Kingdom	6	5	480	80
9	University of California, San Francisco	United States	6	4	273	45.5
10	China Medical University	China	5	4	79	15.8

### 3.4 Journals and co-cited journals

A total of 286 journals published research findings on the relationship between immunity and insomnia, with 4,721 journals being co-cited. Table 3 presents the publication volume and citation metrics of the journals and co-cited journals. Brain, Behavior, and Immunity (Britain, *n* = 12) is the journal with the highest publication volume, followed by Sleep Medicine (Britain, *n* = 10) and Frontiers in Neurology (Switzerland, *n* = 9). Sleep (United States, *n* = 715) is the most frequently co-cited journal. These journals have significantly contributed to the dissemination and sharing of knowledge in the field of immunity and insomnia research.

**Table 3:** The top 10 journals and co-cited journals according to research on immunity and insomnia during 2000–2023.

Rank	Journal	Publication	Citations	Co-cited journals	Co-citations
1	Brain, Behavior, and Immunity	12	1,734	Sleep	715
2	Sleep Medicine	10	181	Neurology	529
3	Frontiers in Neurology	9	117	Brain, Behavior, and Immunity	515
4	BMC Neurology	8	75	PNAS	419
5	Frontiers in Immunology	6	76	Nature	353
6	Medical Hypotheses	6	133	PLOS ONE	348
7	Neurology India	6	43	Annals of Neurology	343
8	Sleep	6	127	Sleep Medicine	304
9	Frontiers in Neuroscience	5	37	Science	275
10	International Journal of Molecular Sciences	5	66	The Lancet	273

### 3.5 Authors and co-citations authors

A total of 2,602 authors have contributed to the research on immunity and insomnia, with 18,663 co-cited authors. Table 4 presents the publication and citation metrics of the authors and co-cited authors. Michael R. Irwin (University of California, *n* = 10) is the most prolific author, followed by Sergio Tufik (Federal University of São Paulo, *n* = 6) and Michael Maes (Chulalongkorn University, *n* = 5). Michael R. Irwin (University of California, *n* = 194) is also the most frequently co-cited author, followed by Alexandros (Penn State College of Medicine, *n* = 113) and Josep Dalmau (University of Barcelona, *n* = 137). These authors have made significant contributions to the exploration of the impact of immunity on insomnia.

**Table 4:** The top 10 authors and co-citations authors according to research on immunity and insomnia during 2000–2023.

Rank	Authors	Publication	Citations	Co-citations authors	Co-citations
1	Michael R. Irwin	10	1,166	Michael R. Irwin	194
2	Sergio Tufik	6	123	Alexandros N. Vgontzas	113
3	Michael Maes	5	164	Josep Dalmau	99
4	Richard Olmstead	5	142	Michael Irwin	76
5	Angela Vincent	5	563	James M. Krueger	74
6	Monica Levy Andersen	4	83	Sarosh R. Irani	69
7	Francesc Graus	4	531	Charles M. Morin	49
8	Andrew McKeon	4	247	Till Roenneberg	48
9	Sean J. Pittock	4	313	Félix Lechin	47
10	Adriano Zager	4	42	Stanley B. Prusiner	47

### 3.6 Keywords

**Figure 5:**
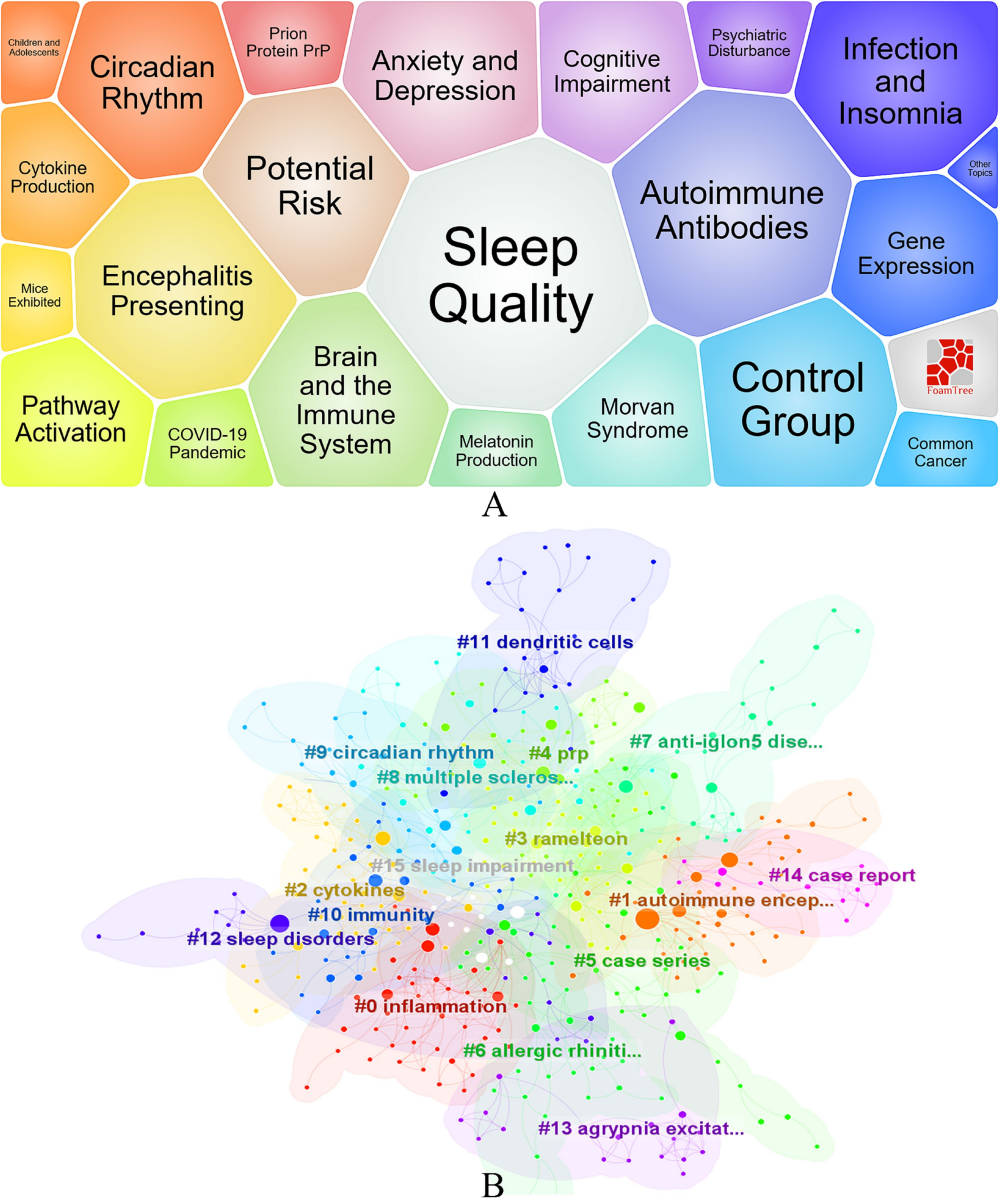
Visualization of Research on Immunity and Insomnia Keywords. **(A)** Keyword Bubble Chart. **(B)** Keyword clustering diagram.

Through systematic organization and analysis of keywords, a total of 1,294 keywords were extracted, revealing the main trends and hotspots in current insomnia research. Insomnia (*n* = 120) is the most frequently occurring keyword, highlighting its central role in the research. Additionally, keywords such as inflammation (*n* = 68), COVID-19 (*n* = 44), and autoimmune encephalitis (*n* = 38) also reflect the recent focus areas in this field. Notably, COVID-19 (*n* = 243) shows a high frequency and strong association, indicating its significant impact on insomnia research. The keyword bubble chart (Figure 5A) and cluster analysis (Figure 5B) further elucidate the core themes and directions of the research. Keywords such as sleep quality, anxiety and depression, and brain and the immune system not only highlight the current research hotspots but also suggest potential future research pathways. Cluster analysis reveals that #0 inflammation, #1 autoimmune encephalitis, #2 cytokines, #9 circadian rhythm, and #11 dendritic cells are key areas of current research, reflecting the in-depth exploration of immune mechanisms in insomnia studies. Although keywords such as “inflammation” and “autoimmune encephalitis” frequently appear, indicating that research has delved into the role of immune mechanisms in insomnia, the related studies still tend to focus on overarching mechanisms, without diving deeply into more specific molecular levels. For example, the precise role of certain cytokines (e.g., IL-6, TNF-*α*) in the development of insomnia may still be underexplored, warranting further investigation.

Additionally, cluster analysis also revealed the appearance of keywords such as “dendritic cells,” suggesting their potential role in immune regulation. However, research in this area is currently limited, and the role of dendritic cells in immune responses, particularly in the pathogenesis of insomnia, has not been adequately explored.

Although the interaction between the biological clock and the immune system has received some attention, the specific mechanisms of this interaction in the context of insomnia remain unclear. Particularly from the perspective of individual differences, investigating how different chronotypes affect immune responses may provide new insights for future precision medicine treatments. In summary, while research on insomnia and its relationship with immunity has made progress, significant gaps remain in areas such as cytokines, dendritic cells, and individual differences. These gaps provide important directions for further exploration in the future.

### 3.7 Co-cited references

**Figure 6:**
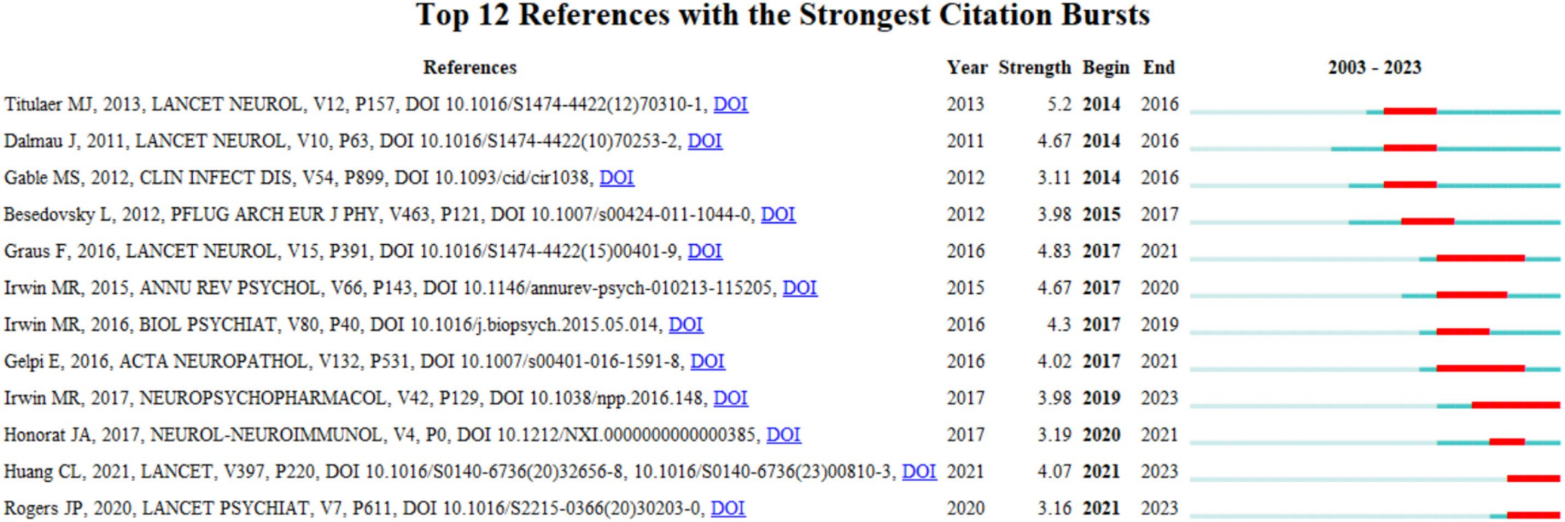
Research on immunity and insomnia co cited literature.

Through an analysis of highly cited co-cited literature, we identify key studies that have significantly advanced the understanding of immune responses in the context of insomnia. Figure 6 highlights the significant impact of three key publications by Maarten J. Titulaer (Strength = 5.2), Francesc Graus (Strength = 4.84), and Josep Dalmau (Strength = 4.68) on immune cell interactions, with a focus on how these findings relate to insomnia research from 2014 to 2018.

The study by Maarten J. Titulaer et al., published in 2013 (13), is a pioneering work in the field of neuroimmunology, exploring the immunotherapeutic effects and long-term prognosis of anti-NMDAR encephalitis. This study significantly influenced clinical practice and was widely cited between 2014 and 2016. Its insights into immune dysregulation and neuroinflammation have relevance to insomnia, as they provide a deeper understanding of how immune system dysfunction can contribute to sleep disorders and neuroinflammatory conditions.

Similarly, the research by Josep Dalmau et al., published in 2013 (14), further advanced the study of anti-NMDAR encephalitis. By focusing on the clinical efficacy of immunotherapy treatments, such as corticosteroids and intravenous immunoglobulin, this study has had a profound impact on neuroimmunology. Its detailed analysis of immune response modulation in autoimmune encephalitis informs the potential role of immune therapies in treating insomnia, especially in cases where sleep disturbances are linked to immune system dysfunction.

Francesc Graus et al.’s research, published in 2016 (15), examined the diagnostic methods for autoimmune encephalitis, proposing a syndrome-based diagnostic approach. This work addressed critical challenges in diagnosing immune-related disorders, which is important for insomnia research, as it sheds light on the diagnostic challenges related to immune-mediated sleep disturbances. By introducing early diagnostic strategies, this study has provided valuable insights for clinicians working on sleep disorders with an immune component, thus enhancing the potential for early intervention and more effective treatment strategies for insomnia.

These studies have not only deepened our understanding of immune cell roles in neuroimmunology but also provided essential references for exploring immune responses in insomnia, a field that is increasingly recognized as playing a key role in the pathogenesis of sleep disorders. By building on these foundational works, current research can further investigate how immune modulation may serve as a therapeutic approach for insomnia.

## 4 Discussion

### 4.1 T cells and insomnia

T cells are the main effector cells in the immune system, divided into CD4+ helper T cells and CD8+ cytotoxic T cells, responsible for regulating and executing immune responses. A study by Junhan Lin et al. (16) found that in patients with sleep disorders, the levels of activated CD4+ T cells and CD8+ T cells were significantly elevated, along with a marked increase in central memory CD4 T cells, central memory CD8 T cells, and natural killer T cells. This suggests that T cells play an important role in the immune response associated with sleep disorders.

Further research by Jiaoshi Zhao et al. (17) showed that a novel electrotherapy method could effectively improve insomnia in patients with autoimmune rheumatic diseases by modulating changes in T cell subsets. This offers a new possibility for immune-based treatments for sleep disorders.

In another study, Mei Rong et al. (18) demonstrated that a modified Qi Yuan ointment could improve the immune function of insomnia mice by regulating the GABA/Glu content and the expression of related proteins. Specifically, it enhanced the activity of CD4+ T cells and suppressed the activity of CD8+ T cells, suggesting that T cells play a crucial role in immune regulation associated with insomnia. This provides new insights into the mechanisms underlying the interaction between insomnia and the immune system.

Moreover, Mihret Melese et al. (19) conducted a systematic review and meta-analysis to explore the relationship between insomnia and CD4+ T cells in HIV/AIDS patients. The study revealed that 49.32% of HIV/AIDS patients in sub-Saharan Africa experienced poor sleep quality. Depression and a CD4 count below 200 cells/mm^3^ were key factors contributing to poor sleep quality. Notably, patients with a CD4 count below 200 cells/mm^3^ had a significantly increased likelihood of poor sleep quality (OR 3.15). This highlights the close association between the reduction of CD4+ T cells and insomnia, emphasizing the urgency of improving sleep quality in HIV/AIDS patients in the context of depression and immune suppression.

These studies collectively reveal the important role of T cells in the immune response related to insomnia. They provide a solid foundation for further understanding the interaction between insomnia and the immune system and offer strong support for future clinical intervention strategies.

### 4.2 B cells and insomnia

B cells are an important component of the immune system, responsible for producing antibodies and regulating immune responses. In recent years, increasing attention has been given to the role of B cells in sleep disorders, particularly insomnia. Insomnia not only affects an individual’s quality of life but may also increase the risk of infections and inflammatory diseases by impacting the immune system. As key regulators of humoral immunity, dysfunction of B cells may play a crucial role in the immune response and chronic inflammation associated with insomnia.

Several studies have revealed the significant impact of circadian rhythm disruption and sleep deprivation on the immune system, especially on B cell function. Research by Hitoshi Inokawa et al. (20) demonstrated that long-term circadian rhythm disruption exacerbates immune system aging and increases the number of CD95 + GL7+ germinal center B cells, which are closely linked to chronic inflammation. Another study found that sleep deprivation suppresses the expression of BMAL1 and CLOCK genes, which in turn inhibits the production of TGF-β1 and reduces the number of IgG2b + B cells (21).

Furthermore, Ben Korin et al. (22) showed that after six hours of sleep deprivation, the number of B cells in the brain significantly increased, which was associated with the upregulation of the B cell migration-related receptor CXCR5 and its ligand CXCL13 in the meninges. Hong Xie et al. (23) assessed changes in various types of immune cells in patients with sleep apnea/hypopnea syndrome and found that these changes were correlated with the severity of oxygen desaturation. Additionally, Lisandro Lungato et al. (24) explored the effects of sleep deprivation on B lymphocytes in the bone marrow and spleen, revealing that sleep deprivation not only reduced the number of B lymphocytes but also increased their mortality rate, accompanied by elevated oxidative stress markers. These findings collectively highlight the multiple effects of sleep disorders on B cell immune function, suggesting their potential role in chronic inflammation and immune aging.

### 4.3 NK cells and insomnia

There exists a complex bidirectional relationship between insomnia and the immune system. Shortened sleep duration is closely associated with increased inflammatory processes and a heightened risk of infection. In patients with insomnia, the number of NK (natural killer) cells is significantly reduced during sleep, which may be due to their migration to secondary lymphoid organs. However, despite the decrease in number, their activity is found to increase (25). In contrast, some studies have found that NK cell levels are significantly elevated in insomnia patients (16).

Moreover, in liver cancer patients, those with insomnia show significantly lower NK cell levels, which contrasts sharply with findings from other studies (26). In patients receiving adjuvant endocrine therapy for breast cancer, supplementation with Reishimmune-S (a fungal immune-regulating peptide) significantly improved cognitive function and reduced fatigue and insomnia levels. After 6 months of Reishimmune-S supplementation, the composition of NK cell subsets changed, with a notable decrease in the proportion of NKG2A+ and NKp30+ NK cells. Additionally, the degree of fatigue was positively correlated with the proportion of NKp30+ NK cells (27). These findings suggest that changes in NK cell subsets may be closely related to insomnia and fatigue.

Further research has shown that in both insomnia and myocardial infarction patients, the downregulation of resting NK cells is positively correlated with the expression of the SYTL2 gene. The SYTL2 gene is closely involved in multiple NK cell signaling pathways, including the MAPK signaling pathway, the movement of cytotoxic granules, exocytosis, and NK cell activation. Additionally, SYTL2 is involved in regulating NK cell-mediated cytotoxicity and immune responses (28). These studies collectively suggest that NK cells may play a key role in the relationship between insomnia and immune function. Changes in their subsets and the expression of related genes are closely associated with the pathological mechanisms of insomnia.

### 4.4 Neutrophils and insomnia

There is a complex interaction between insomnia and neutrophil-mediated inflammatory responses. Research indicates that insomnia may lead to a significant increase in neutrophil numbers in peripheral blood. For example, mice subjected to prolonged sleep deprivation show an accumulation of neutrophils in the circulation, which may be due to the inflammatory response induced by sleep deprivation, stimulating the bone marrow to release more neutrophils into the bloodstream (29). Additionally, insomnia may affect neutrophil function, enhancing their activity. This is reflected in the release of more inflammatory mediators and extracellular traps (NETs), but their normal immune defense function may be impaired, leading to a reduced ability to resist pathogens (30).

Conversely, neutrophil-mediated inflammatory responses also influence sleep. Sleep deprivation induces an increase in prostaglandin D2 (PGD2) levels in the brain. PGD2 crosses the blood–brain barrier and leads to the accumulation of circulating neutrophils and a cytokine storm-like syndrome. This inflammatory response not only causes systemic inflammation but may also trigger multiple organ dysfunction syndrome (MODS), further exacerbating insomnia symptoms (29). Furthermore, the activation of neutrophils and the increase in inflammatory factors are positively correlated with fatigue and insomnia levels in patients with sleep disorders (31).

Therefore, intervening in neutrophil-mediated inflammatory responses may be a key approach for treating recurrent insomnia. With pharmacological intervention, lifestyle adjustments, or immune regulation therapies, it is hoped that symptoms of insomnia in patients can be improved, leading to better quality of life.

### 4.5 Monocytes and insomnia

Monocytes play an important role in the onset and progression of insomnia. Insomnia may lead to an increase in monocyte numbers and functional changes, characterized by enhanced pro-inflammatory activity and the secretion of more inflammatory cytokines, such as interleukin-6 (IL-6) and tumor necrosis factor-alpha (TNF-*α*) (32). These inflammatory cytokines activate the NF-κB signaling pathway, further exacerbating systemic inflammation and potentially affecting sleep quality, creating a vicious cycle. Additionally, insomnia may further enhance the pro-inflammatory function of monocytes by activating the sympathetic nervous system and the NLRP3 inflammasome (33).

Anti-inflammatory treatments, lifestyle modifications, and the use of immune modulators, such as melatonin, may help alleviate insomnia symptoms by inhibiting the production of inflammatory cytokines and regulating monocyte function (34). Therefore, intervening in monocyte-mediated inflammatory responses is one of the key approaches to treating insomnia. Future research needs to further explore the specific mechanisms of monocyte involvement, providing a theoretical basis for the development of new treatment strategies.

### 4.6 Comorbidities of insomnia

#### 4.6.1 Cardiovascular diseases

In recent years, the comorbid relationship between insomnia and atherosclerosis has garnered widespread attention. Research has shown that insomnia patients often exhibit a chronic inflammatory state, with significant changes in the quantity and function of immune cells (such as monocytes and macrophages) in peripheral blood, along with increased secretion of inflammatory cytokines (such as interleukin-6 and tumor necrosis factor-alpha) (31). These factors play a crucial role in the formation and instability of atherosclerotic plaques. Furthermore, insomnia may exacerbate the inflammatory response by affecting the metabolic state of immune cells, leading to endothelial cell damage and increased immune cell infiltration, which can compromise plaque stability (35).

Recent studies have further elucidated how sleep regulates hematopoiesis, influencing immune cell production and contributing to atherosclerosis. Specifically, sleep fragmentation has been shown to increase the production of Ly-6Chigh monocytes, which are linked to the development of larger atherosclerotic lesions. The research conducted by Cameron S. McAlpine et al. (36) demonstrates that the neuropeptide hypocretin, which plays a pivotal role in regulating myelopoiesis, is crucial in this process. Sleep fragmentation leads to a reduction in hypocretin levels, resulting in increased monocyte production and accelerated atherosclerosis. However, supplementing hypocretin or modulating CSF1 production, a key regulator of myelopoiesis, can reduce monocyte levels and help slow the progression of atherosclerotic lesions.

Clinical studies have found that patients with insomnia have significantly elevated levels of high-sensitivity C-reactive protein (hs-CRP), which is closely associated with the severity of atherosclerosis and the incidence of cardiovascular events. Additionally, insomnia may further worsen inflammation by influencing the metabolic state of immune cells. For example, prolonged sleep deprivation leads to irreversible changes in the DNA structure of immune cells, and even after sleep is restored, these immune cells retain epigenetic marks of inflammatory activation, making the body more prone to inflammatory diseases and cardiovascular conditions (37).

Given this, improving sleep quality and modulating immune cell inflammatory responses may become an important strategy for improving cardiovascular disease prognosis. Future research can further explore the specific mechanisms between insomnia, atherosclerosis, and hypertension, and develop integrated treatment plans to provide more effective therapeutic options for cardiovascular patients.

#### 4.6.2 Obesity

Insomnia and insufficient sleep are closely linked to weight gain, obesity, and the onset of related metabolic diseases. Prolonged sleep deprivation causes irreversible changes in the DNA structure of immune cells, making the body more susceptible to inflammatory diseases and metabolic disorders (38). In patients with obesity, there is an increase in pro-inflammatory immune cells in peripheral blood (such as monocytes and macrophages), elevated levels of inflammatory cytokines (such as IL-6 and TNF-*α*), and a decrease in regulatory T cells in adipose tissue, while pro-inflammatory T cells increase. This immune imbalance may lead to chronic inflammation in adipose tissue and insulin resistance (39).

Furthermore, the circadian rhythms of immune cells are regulated by sleep, and sleep deprivation disrupts these rhythms, leading to immune system dysfunction, which further exacerbates the development and progression of obesity (40). Therefore, improving sleep quality and modulating immune cell function may become an important strategy for improving obesity and its related metabolic diseases. Future research can further explore the specific mechanisms behind this and develop comprehensive treatment plans to address these health concerns.

#### 4.6.3 Depression

Peripheral immune cells and central immune cells play a crucial role in the comorbidity of depression and insomnia. Depression is often accompanied by sleep disorders, which are closely related to dysfunction in the brain’s waste clearance system. Studies have shown that the global blood oxygen level-dependent signal (gBOLD) coupling strength with cerebrospinal fluid (CSF) flow dynamics is weakened in depressed patients compared to healthy controls, which may be due to damage to the glymphatic system. Additionally, in depressed patients, gBOLD-CSF coupling strength correlates positively with mid-phase insomnia (41). These results suggest that defects in the brain’s waste clearance system in depressed patients may be closely related to their decreased sleep quality.

Furthermore, peripheral blood immune cells, especially blood cell counts, in depressed patients are significantly associated with the severity of depressive symptoms and their various dimensions. Research has shown that white blood cell count is significantly correlated with the “pathological” dimension (including anhedonia, psychomotor retardation, fatigue, and loss of appetite), particularly in male patients. Platelet count, on the other hand, is significantly associated with the “insomnia/agitation” dimension (including early, middle, late insomnia, and agitation) and suicidal ideation, especially in female patients (42). These findings suggest that white blood cells may be related to the pathological symptoms of depression, while platelets may be associated with insomnia, agitation, and suicidal ideation. However, these associations do not necessarily imply causality.

This highlights the complex relationship between immune cell function, sleep disorders, and depressive symptoms, suggesting the need for further investigation into how immune system dysfunction may contribute to the onset and progression of depression and its comorbid conditions.

#### 4.6.4 Cancer

Sleep plays a critical role in regulating immune system function, influencing the quality and duration of immune responses, and thereby having significant implications for tumor progression and prevention. The immune system plays a key role in cancer prevention and progression, primarily through immune surveillance mechanisms and regulation of the tumor microenvironment, which can affect cancer initiation and development. However, sleep disorders such as insomnia may impair immune surveillance, promote tumor progression, and influence cancer prognosis (43).

Moreover, research indicates that insomnia and physiological stress can disrupt the body’s balance mechanisms by affecting the hypothalamic–pituitary–adrenal (HPA) axis and sympathetic-adrenal-medullary (SAM) axis. The interaction between immune cells and the vaginal microbiome, particularly through changes in cytokine profiles and modulation of related immune capabilities, can impact cervical bioactivity. These immune mechanisms, in combination with insomnia and physiological stress, may exacerbate the onset of cervical cancer (44).

This suggests that the relationship between sleep, immune function, and cancer is multifaceted. Disruptions in sleep, particularly insomnia, could have a profound impact on immune responses that help control cancer development, highlighting the need for better understanding and management of sleep disorders in cancer prevention and therapy.

### 4.7 Limitations

#### 4.7.1 Data source limitations

The data for this study were sourced from the Web of Science Core Collection (WoSCC) database, while other significant databases such as PubMed and Scopus were not included. This may have led to the omission of some relevant literature, and as a result, the findings may not fully reflect the entire scope of research on the relationship between immunity and insomnia.

#### 4.7.2 Limitations of literature screening criteria

Although the literature screening criteria were clearly defined, there is potential for subjective bias due to the reliance on manual screening. Additionally, the exclusion of conference papers and non-English literature might have resulted in the omission of important studies, particularly in research fields from non-English-speaking countries.

## 5 Conclusion

This study systematically reveals the current status and development trends of immunological insomnia research through a bibliometric analysis of literature published between 2000 and 2023 on the relationship between the immune system and insomnia. The results indicate that immune cells, particularly T cells, B cells, NK cells, neutrophils, and monocytes, play an important role in the pathogenesis of insomnia. These immune cells not only participate in the onset of sleep disorders but also form a complex interaction between the immune system and sleep disturbances. Recent research suggests that immune cells play a key role in regulating the nervous and endocrine systems, especially in inflammation and immune modulation. Future in-depth exploration of the functions of these immune cells will help uncover the immunological basis of insomnia and provide new directions for the development of related therapeutic strategies.

Moreover, this study also found that several comorbidities, including cardiovascular diseases, obesity, depression, and cancer, are closely associated with immune insomnia. Abnormal immune responses may not only lead to the onset of sleep disorders but also exacerbate the progression of these chronic diseases. Therefore, research on immunological insomnia is not only important for understanding insomnia itself but also holds potential clinical value in the prevention and treatment of these comorbidities.

As research on insomnia advances, single-disciplinary research methods are no longer sufficient to meet the needs of exploring its complex mechanisms. Future research should encourage multidisciplinary collaboration and integrate the strengths of immunology, neuroscience, clinical medicine, psychology, and other fields. Only through interdisciplinary cooperation can we more comprehensively understand the complex interactions between the immune, nervous, and endocrine systems, thereby providing more systematic and comprehensive solutions for the early diagnosis and precise treatment of immune insomnia.

However, this study also has certain limitations. First, the research is based solely on data from the Web of Science Core Collection (WoSCC), which may miss relevant literature from other databases. Additionally, the exclusion of conference papers and non-English literature may result in some research findings being excluded from the analysis.

In conclusion, the interaction between immune cells and insomnia has become a hot topic in current research. Future studies should continue to explore the multifaceted role of the immune system in the onset of insomnia, particularly how regulating immune responses can improve sleep quality. Attention should also be given to the association between immune insomnia and various comorbidities. More importantly, by strengthening interdisciplinary collaboration and combining research from different fields, more precise and personalized treatment strategies can be developed for the treatment of immune insomnia.
